# Mechanical Properties of Dental Enamel in Patients with Genetic Caries Susceptibility

**DOI:** 10.3390/ijms26167749

**Published:** 2025-08-11

**Authors:** Firas Haj Obeid, Karolina Jezierska, Danuta Lietz-Kijak, Piotr Skomro, Totka Bakalova, Jacek Gronwald, Piotr Baszuk, Cezary Cybulski, Wojciech Kluźniak, Barbara Gronwald, Magdalena Sroczyk-Jaszczyńska, Alicja Nowicka, Petr Louda, Helena Gronwald

**Affiliations:** 1 Department of Propaedeutic, Physical Diagnostics and Dental Physiotherapy, Faculty of Medicine and Dentistry, Pomeranian Medical University in Szczecin, 70-204 Szczecin, Poland; firashajobeid@wp.pl (F.H.O.); danuta.lietzkijak@gmail.com (D.L.-K.); piotr.skomro@pum.edu.pl (P.S.); 2 Department of Medical Physics, Pomeranian Medical University in Szczecin, 71-073 Szczecin, Poland; karolina.jezierska@pum.edu.pl; 3 Department of Material Science, Technical University of Liberec, 461 17 Liberec, Czech Republic; totka.bakalova@tul.cz; 4 Department of Genetics and Pathology, Pomeranian Medical University in Szczecin, 71-252 Szczecin, Poland; jacek.gronwald@pum.edu.pl (J.G.); piotr.baszuk@pum.edu.pl (P.B.); cezary.cybulski@pum.edu.pl (C.C.); wojciech.kluzniak@pum.edu.pl (W.K.); 5 Doctoral School, Pomeranian Medical University in Szczecin, 71-210 Szczecin, Poland; barbara.gronwald@pum.edu.pl; 6 Department of General, Dental and Interventional Radiology, Pomeranian Medical University in Szczecin, 70-111 Szczecin, Poland; magdalena.sroczyk.jaszczynska@pum.edu.pl; 7 Department of Conservative Dentistry and Endodontics, Faculty of Medicine and Dentistry, Pomeranian Medical University in Szczecin, 70-111 Szczecin, Poland; nowicka6@gmail.com; 8 NovoDental Clinic, 71-219 Bezrzecze, Poland; 9 Faculty of Engineering, University of Kalisz, 62-800 Kalisz, Poland; petrlbc1@seznam.cz

**Keywords:** enamel biomineralization, formation, mechanical properties, material science, polymorphism(s), caries detection, diagnosis, prevention

## Abstract

This study evaluated the physicochemical and morphological properties of tooth enamel in patients with caries-predisposing SNPs (rs4694075 in *AMBN* and rs2337359 in *TUFT1* genes), based on the DMFT index. We included 40 of 120 individuals (aged 19–43), collecting stimulated saliva and 58 healthy teeth extracted for orthodontic/surgical reasons. Saliva DNA was genotyped. Enamel properties were assessed using Vickers microhardness, deposition thickness, and calcium content. Genotype and allele frequencies aligned with the literature. The *TUFT1*C/C genotype subgroup showed a significantly higher DMFT index (*p* = 0.03) compared to the T/T genotype, while *AMBN* showed no such correlation. Calcium content, microhardness, and enamel thickness were similar across all polymorphic variants of both genes. A statistically significant correlation (*p* = 0.003) was found between reduced enamel calcium content and a higher DMFT index. Despite existing literature on the subject, the studied SNPs did not reflect any correlation with morphological or physicochemical changes in enamel. The above results suggest that genetic variability identifies patients classified by dentists as being at higher risk of caries, even though these patients follow a non-cariogenic diet and adhere to a hygiene regime. As no structural or physicochemical changes in the enamel of this group were observed, the potential cause may be disturbances in the remineralisation mechanisms or enamel surface properties that promote biofilm adhesion in polymorphic patients. Intensive tooth calcification control algorithms using LIF and RVG, as well as remineralisation cycles to increase hydroxyapatite saturation with calcium phosphates and bioadhesive fluoride delivery systems for long-term biofilm control, are used to more effectively prevent or slow down the progression of caries.

## 1. Introduction

Dental caries is one of the foremost challenges in modern dentistry, and its etiopathogenesis has been extensively studied [1,2]. Numerous studies have highlighted the correlation between specific polymorphic variants and heightened susceptibility to caries [3,4,5,6]. Studies on human teeth in vitro [3] have shown that the genes responsible for enamel formation influence its microhardness following a cariogenic challenge. This is crucial for understanding the genetic factor that influences tooth resistance to caries. A study of asthmatic children in Turkey [4] has confirmed an increased risk of tooth decay in this population due to polymorphism in the ameloblastin gene. This gene variation is important for understanding the role of genetics in the aetiology of tooth decay in chronically ill patients. In their literature review [5], the authors concluded that the risk of caries in patients is the result of a complex interaction between genetic and environmental factors, which highlights the need for a personalised approach to prevention. Another review article [6] states that genetics influences tooth decay through enamel development, saliva composition, and immune response. This means that it must also be prevented in many ways. Candidate gene studies for caries susceptibility typically include four main types of genes: genes involved in enamel development, saliva composition, immune response, and taste perception [7]. This study of 80 Caucasian individuals aged 21 to 32 showed that variations in the *GLUT2* and *TAS1R2* genes may be linked to a higher caries risk. This is significant as it helps us understand the genetic basis of sweet taste perception and its effect on caries resistance. The following study focuses on genes involved in enamel development, i.e., *AMBN* and *TUFT1*, encoding ameloblastin and tuftelin, respectively.

Ameloblastin, a key component of the enamel matrix, maintains ameloblast differentiation during the secretory phase of enamel formation. At the same time, tuftelin contributes to enamel crystal formation by predominating in the enamel bundles, the less mineralised regions [8,9,10,11]. Fukumoto et al. (2005) [8] present the key role of ameloblastin in ameloblast differentiation and enamel formation. Mazumder’s study (2016) on mice [9] demonstrated the spatial interactions between amelogenin and ameloblastin proteins during enamel maturation. This is the essence of the molecular mechanisms of enamel formation. Berkovitz et al. (2017) [10] presented the details of tooth histology and embryology, which form the basis for any study of the structure and properties of enamel. In a meta-analysis and systematic review, Sharifi et al. (2021) [11] showed that polymorphisms in the *CA VI*, *AMBN*, and *TUFT1* genes are associated with an increased risk of dental caries, providing strong evidence of a genetic basis for the disease.

The selected SNPs are located in the intron regions (rs2337359-chr1:151523320) and (rs4694075-chr4:70601197). While studies have suggested their association with caries susceptibility [5,12,13], none have directly linked genetic predisposition to enamel morphology and material properties. The study by Shaffer et al. (2015) [12] used data from 3600 participants from five populations and showed that exposure to fluoride moderates the effect of enamel matrix genes on susceptibility to caries. The results of the Piekoszewska-Ziętek (2017) study [13] found that SNPs in genes linked to enamel development and saliva composition contribute to caries risk. This study’s significance: it provides strong evidence of genetic indicators of disease risk, identifying specific genes (*AMELX*, *AQP5*, *ESRRB*).

Previous studies have mainly focused on elevated DMFT (decayed, missing, and filled permanent teeth index) values as the outcome of caries progression in individuals with predisposing polymorphic variants [3,6,10,11]. Identifying the mechanisms of caries risk associated with specific genetic alterations could improve dental care for patients with an increased risk of caries that is not due to a cariogenic diet or poor oral hygiene. These patients may be offered intensive tooth calcification control algorithms using LIF and RVG, as well as preventive and remineralising cycles (using CPP-APP) to increase the saturation of hydroxyapatite with calcium phosphates and/or the use of bioadhesive delivery systems (such as liposomal fluoride carriers, LCS, hydrogels) to prolong contact with the tooth surface, which is ultimately crucial for biofilm control.

Therefore, this study aims to fill the gap in knowledge on the relationship between enamel morphology, physicochemical properties, genetic predisposition, and susceptibility to caries with clinical consequences. To the best of our knowledge, this is the first study to directly analyse these links.

**Figure 1:**
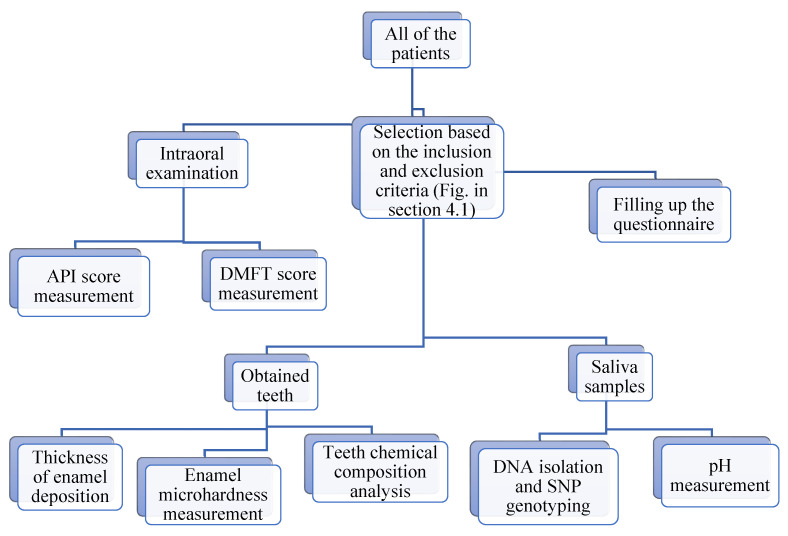
Study design.

This study aims to assess the morphological and physicochemical properties of enamel correlated with the DMFT index in patients with caries-predisposing polymorphic variants in the genes responsible for enamel formation. Study design—see Figure 1.

## 2. Results

### 2.1. Frequency of Genotypes and Alleles Among Participants

**Figure 2:**
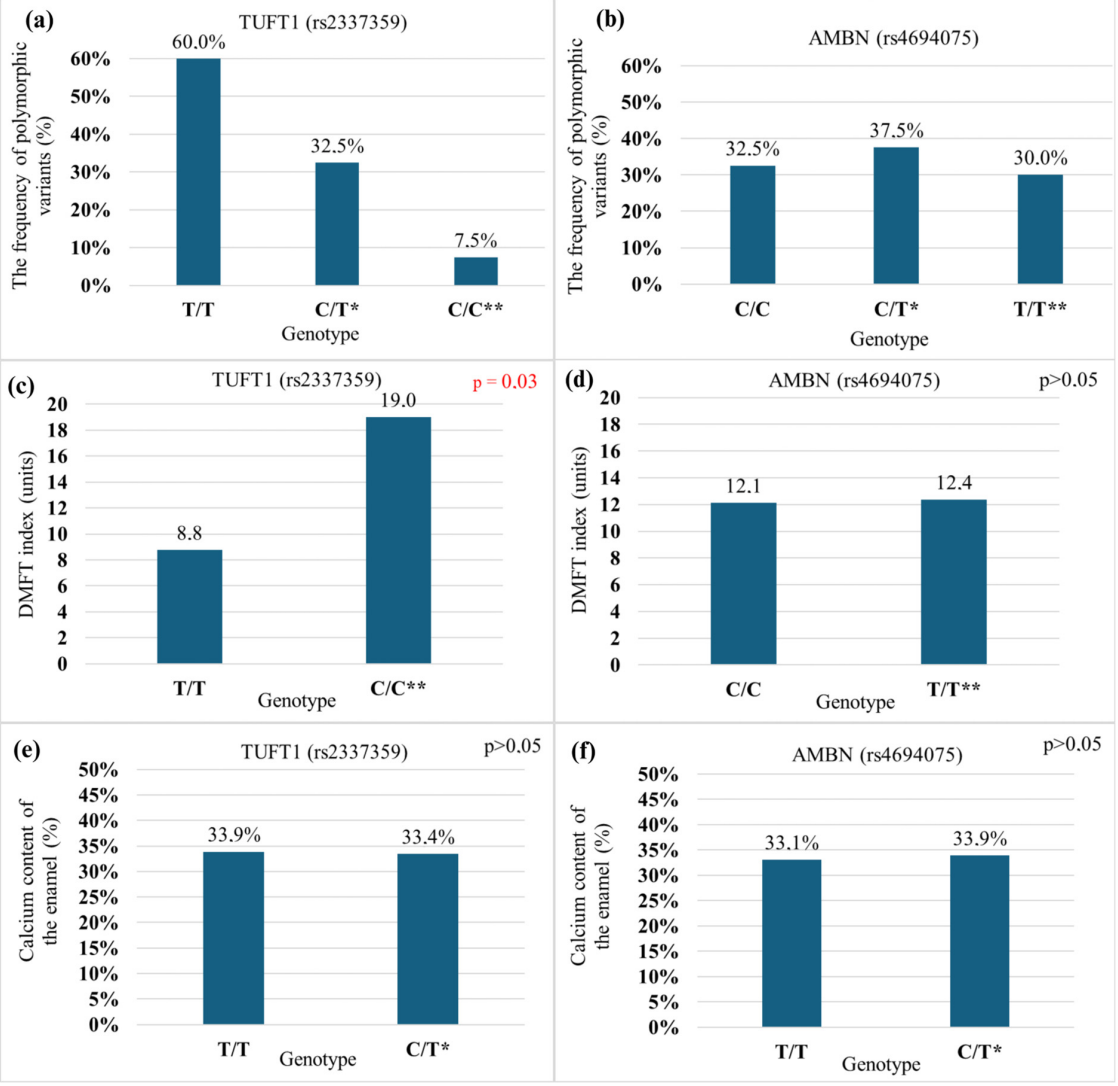
The genotype structure of the participant group related to the tested SNPs TUFT1 rs2337359 (**a**) and AMBN rs4694075 (**b**) (*—indicates the presence of one allele associated with increased caries susceptibility, while ** denotes the presence of two alleles predisposing to caries). DMFT index values in patients with polymorphic variants of the TUFT1 gene rs2337359 (**c**) and the AMBN gene rs4694075 (**d**). Enamel calcium content (%) in the polymorphic variants of the TUFT1 rs2337359 gene (**e**) and the AMBN rs4694075 gene (**f**) studied. Note: from the qualified teeth for chemical composition analysis, none belonged to patients with C/C genotype.

The data revealed that for rs2337359 (*TUFT1*), the most common allele was T = 0.774, while for rs4694075 (*AMBN*), it was C = 0.57. These frequencies closely resembled those reported in the literature (rs2337359: T = 0.819, C = 0.181; rs4694075: T = 0.490, C = 0.501) [14]. Analysis of rs2337359 genotypes showed that only 7.5% of participants had the C/C genotype, which is associated with higher caries susceptibility. The distribution of rs4694075 genotypes was almost equal among participants (see Figure 2a,b).

### 2.2. DMFT Index Value in Patients with TUFT1 (rs2337359) and AMBN (rs4694075) Gene Polymorphic Variants (n = 80)

A statistically significant difference in DMFT values was observed in polymorphic variants of the *TUFT1* gene rs2337359. The group without caries-predisposing alleles (T/T) exhibited lower DMFT values than the group with both caries-predisposing alleles (C/C), which showed the highest DMFT values (*p* = 0.03) (see Figure 2c).

For polymorphic variants of the rs4694075 of the *AMBN* gene, there were no statistically significant differences in DMFT values across the study groups (see Figure 2d).

### 2.3. Enamel Calcium Content in Patients with TUFT1 rs2337359 and AMBN rs4694075 Gene Polymorphic Variants (n = 34)

The enamel calcium content was similar across all polymorphic variants of the *AMBN* and *TUFT1* genes (see Figure 2e,f).

### 2.4. Enamel Microhardness in Patients with Polymorphic Variants of the TUFT1 rs2337359 and AMBN rs4694075 Genes (n = 400)

**Figure 3:**
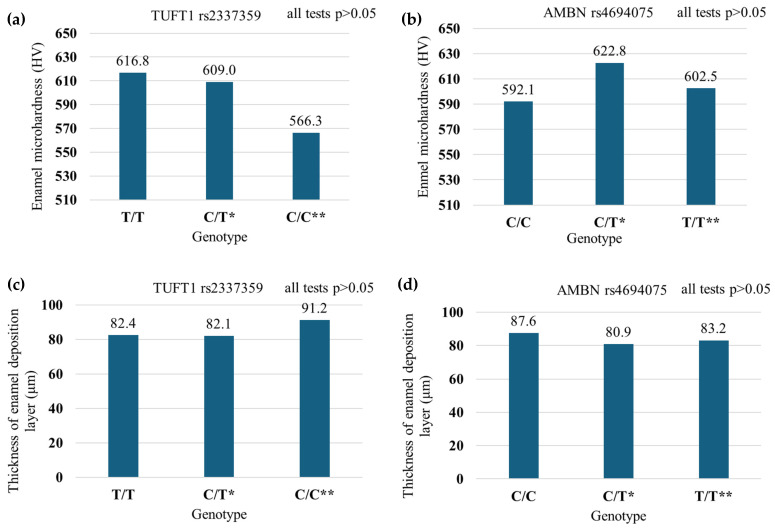
Enamel microhardness (HV) for the tested polymorphic variants of the TUFT1 gene rs2337359 (**a**) and the AMBN gene rs4694075 (**b**). (*—indicates the presence of one allele associated with increased caries susceptibility, while ** denotes the presence of two alleles predisposing to caries). Enamel apposition layer thickness (μm) for the tested polymorphic variants of the TUFT1 gene rs2337359 (**c**) and the AMBN gene rs4694075 (**d**).

There were no statistically significant differences in microhardness for all polymorphic variants of the *AMBN* and *TUFT1* genes (see Figure 3a,b).

### 2.5. The Thickness of the Enamel Apposition Layer in Patients with Polymorphic Variants of the TUFT1 (rs2337359) and AMBN (rs4694075) Genes (n = 400)

The thickness of the enamel apposition layer for all polymorphic variants of the *AMBN* and *TUFT1* genes was similar (see Figure 3c,d).

### 2.6. Correlation Between DMFT Index Values and Enamel Calcium Content

**Figure 4:**
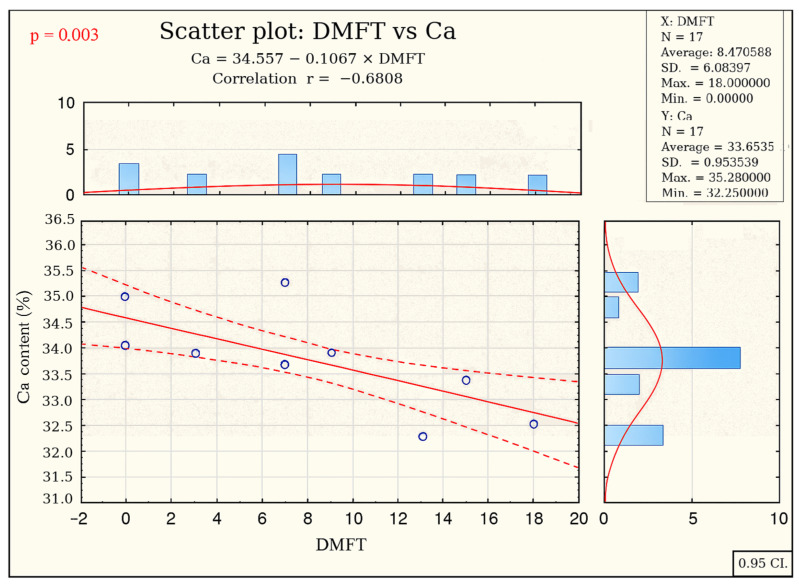
Correlation between a decrease in enamel calcium content and a higher DMFT index value.

We observed a statistically significant (*p* = 0.003) correlation between a decrease in enamel calcium content and a higher DMFT index value (see Figure 4).

### 2.7. Summary of Results

The frequencies of genotypes and alleles for both polymorphisms were consistent with the literature data. The C/C genotype in the *TUFT1* rs2337359 polymorphism was found to be a risk factor for dental caries, as indicated by DMFT values in the study population. However, the *AMBN* rs4694075 polymorphism was not associated with caries susceptibility in the study population based on DMFT values. The study of *TUFT1* rs2337359 and *AMBN* rs4694075 polymorphisms did not reflect the correlation with morphological and physicochemical changes in the enamel, which were expected based on the DMFT index in the scientific literature. Decreased enamel calcium content predisposes patients to a higher risk of caries and requires special dental care.

## 3. Discussion

Our study reveals that *AMBN* rs4694075 SNP variants do not correlate with elevated DMFT values. This contrasts with prior research [3,4,5,15] that linked *AMBN* rs4694075 to caries experience or heightened caries risk. The study by Shimizu et al. (2012) [3] did not assess clinical caries but only changes in enamel microhardness after artificial cariogenic challenge, while other studies [4,5,15] examined DMFT in different populations (including Egyptian and Turkish). However, the 2021 study by Sharifi et al. [11] indicates no association between caries risk and *AMBN* polymorphisms, which is consistent with our findings. It is worth noting that, due to caries’ multifactorial nature, SNPs, especially those in non-coding gene sequences, even though they may influence gene expression, have minimal clinical impact compared to hygiene habits, diet, or socioeconomic status. Consequently, detecting their influence on DMFT values is difficult, even with sophisticated tools. The literature underscores the crucial role of the ameloblastin gene in enamel formation; substantial mutations lead to severe enamel malformations like hypoplasia or hypomineralisation [16]. Conversely, minor gene sequence alterations, including SNPs, may affect protein quality or gene expression, but their presence alone may not designate a patient at higher caries risk, particularly with good hygiene practices, regular check-ups, and a low-carcinogenic diet. Furthermore, ethnicity may influence SNP relevance; variants associated with increased caries risk in Asians may not apply to Caucasians [3,4,5]. 

In the present study, a significant correlation was found between the presence of the C/C genotype in the rs2337359 *TUFT1* gene and increased DMFT index values, which is consistent with previous studies [12,17]. However, this was not reflected in altered enamel microstructure, calcium content, or microhardness. The study by Gerreth et al. (2017) [17] confirms the genetic basis of caries in the Polish population, but in a different age group (children), the differences in the results are because a different polymorphism, rs34538475 in the *AMBN* gene, is analysed, which in turn confirms the complexity of the genetics of caries. It can be assumed that the influence of a given polymorphism on the risk of caries may depend on the stage of tooth development (deciduous vs. permanent teeth) or a different methodology (our study combines clinical data—DMFT, genetic, and laboratory data—microhardness, calcium content, enamel thickness, and the study by Gerreth et al. It is a typical “case-control” association study, without analysis of the physicochemical properties of enamel.)

To detect the influence of genes such as *AMBN*, Shaffer et al. (2015) [12] suggest considering gene–environment interactions, including fluoride. Our results did not show any association with *AMBN* due to the lack of such interactions in the study population or minimal/uniform environmental influences.

It remains unclear how the C/C genotype adversely affects enamel development and, if so, which enamel properties are impaired. In 2012, Shimizu [3] observed reduced enamel microhardness with the presence of the C allele after artificial caries formation, suggesting that caries susceptibility may be due to increased caries progression rather than increased initiation. Enamel demineralisation is recognised as the first stage of caries initiation, which leads to initial caries. Progression of the lesion involves cycles of dissolution and re-deposition of minerals into the hard tooth tissue [18]. Over time, these processes can lead to a net loss of minerals, contributing to the development or progression of carious lesions [18]. The results of the present study confirm the above, showing a correlation between lower enamel surface calcium content and higher DMFT index values in patients, highlighting the importance of calcium for hydroxyapatite formation. Patients with less mineralised enamel are more susceptible to developing caries. This highlights the need for targeted dental care involving monitoring changes in enamel mineralisation and replenishing it when necessary.

We have identified three possible mechanisms that increase the risk of caries in patients with genetic alterations who maintain a healthy diet and good oral hygiene.

The first is structural and physicochemical changes in the enamel, the second is disturbances in the remineralisation mechanisms, and the third is disturbances in the interaction between the enamel surface and the oral environment, hindering biofilm control. Based on the study, it was concluded that the first mechanism is unlikely to be the cause of changes in DMFT. Further research should focus on another potential mechanism, the process of demineralisation and subsequent remineralisation. The third potential mechanism would require examination of the quality of the enamel surface, not only in terms of its geometric structure, but also its susceptibility to fluoride binding. Identification of the mechanisms in specific genetic disorders would allow personalised dental care, in which prevention or treatment is optimised according to the causal mechanism.

Since we are not yet able to identify which mechanism, the second or the third, is most important in patients with the genetic disorders studied, we can offer them more intensive algorithms for examining tooth calcification using FIL and RVG, remineralisation prophylaxis through the use of CPP-ACP to increase the saturation of hydroxyapatite with calcium phosphates, and the use of bioadhesive delivery systems such as liposomal fluoride carriers, LCS, and hydrogels to enable prolonged contact with the tooth surface, which is crucial for biofilm control. 

The hypothesis proposed to explain that the phenomena we observed justify continuing research, not only with a larger group but also with a more extensive cognitive scope. Given the scarcity of literature on the association between material properties of enamel and polymorphic variants of genes related to caries susceptibility, it is worth emphasising that in the present study, thanks to the restrictive selection of material to limit the effects of environmental factors (hygienic, dietary, behavioural, etc.), it was possible to observe the influence of genetic factors. However, the sample size (N = 40) is limited, especially given that ethnic homogeneity of the population is required when using the DMFT index. Therefore, the present study was limited to patients from the Kujawsko-Pomorskie voivodeship in Poland. DNA was extracted from saliva, which made it difficult to obtain good-quality DNA, and this also resulted in a reduction in the study group.

This work highlights the complex interaction between morphological factors and the physicochemical properties of enamel with polymorphic gene variants. A deeper understanding of these relationships could help identify the mechanisms behind increased caries susceptibility associated with certain SNP variants. Future research may lead to the development of targeted dental care strategies.

## 4. Materials and Methods

### 4.1. Study Group

**Figure 5:**
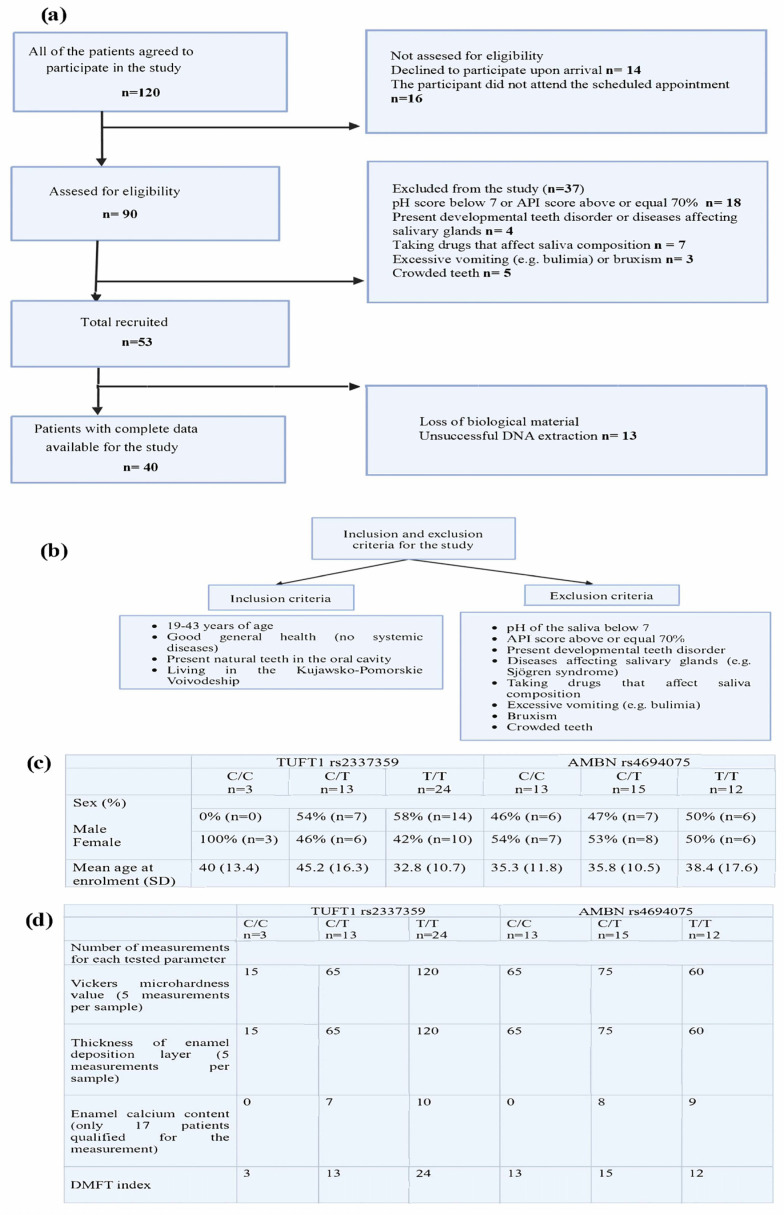
Recruitment of study participants and biological material (**a**). Inclusion and exclusion criteria for the study (**b**). Study groups structure (**c**). Number of measurements for each tested parameter (**d**).

The studied group consisted of subjects from the population of the Kujawsko-Pomorskie voivodeship in Poland, who were selected based on predefined inclusion and exclusion criteria (see Figure 5). 

The inclusion criteria comprised individuals aged between 19 and 43 years who demonstrated good overall health and the absence of systemic diseases, and who possessed natural teeth within the oral cavity. The exclusion criteria were designed to ensure the study’s rigor; they included conditions such as salivary pH below 7, an API score of 70% or more, developmental tooth disorders, diseases affecting the salivary glands like Sjögren’s syndrome, medications that influence saliva composition, excessive vomiting (e.g., bulimia), bruxism, and crowded teeth.

The study was approved by the Bioethics Committee of Pomeranian Medical University (under No. KB-0012/88/17), and all participants gave their consent individually and signed the terms of informed consent. The study involved 40 patients (N = 40) selected from a total of 120 participants. A selection process was carried out with rigorous standards. This process was based on a completed questionnaire, the Approximal Plaque Index (API), and saliva pH measurements taken during the study. Patients scheduled to undergo tooth or teeth extraction for surgical, periodontal, or orthodontic reasons at the Dentus Plus Dental Centre in Bydgoszcz formed the subjects of the study.

### 4.2. Subjective Examination

The participants completed an anonymous questionnaire during the examination. This consisted of questions about their age and gender, as well as any existing medical conditions or medications that might affect saliva production. The survey also covered dietary preferences, oral hygiene practices, and nicotine use.

### 4.3. Physical Examination

During intraoral examinations, the DMFT index and an objective assessment of oral hygiene using the API were recorded for each patient. The documentation was checked to ensure that no fluoridation had been performed. The pH of the stimulated saliva samples was measured using an electronic pH meter (pH Check, Dostmann, Germany).

### 4.4. Collection of Biological Samples

Two millilitres of stimulated saliva were collected from each patient and stored at −20 °C until genotyping. The inclusion of extracted teeth in the study was predicated on the criterion of having at least half of the crown with intact enamel and dentin. These teeth were stored at 4 °C in a 2% sodium hypochlorite solution and rinsed with demineralised water before examination. A total of fifty-eight permanent teeth were included in the study.

### 4.5. Methods of Examination

#### 4.5.1. DNA Extraction, SNP Genotyping, and Patient Grouping

**Figure 6:**
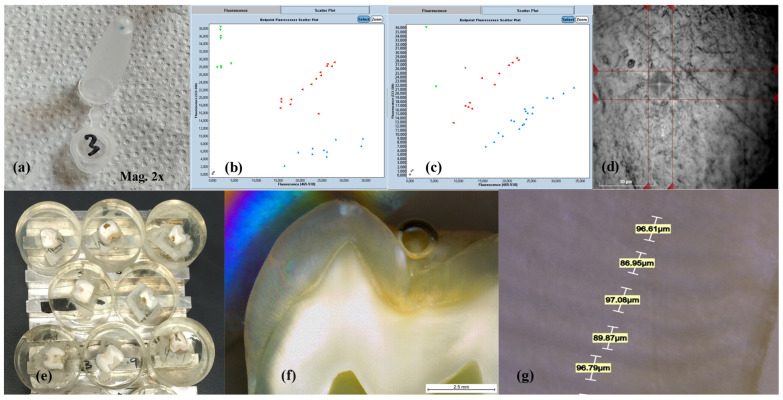
Blue DNA precipitate extracted from one of the saliva samples tested (**a**). Fluorescence distribution of the tested SNPs AMBN (**b**) and TUFT1 (**c**), which were used to determine the patient’s genotype. Note: In the graphs shown, some samples that were not successfully genotyped (coloured grey) were successfully genotyped on the next attempt. Image of an indentation in the enamel made with a Duramin-48 microhardness tester using the Vickers method (**d**). Samples of tooth enamel embedded in epoxy resin (**e**). Images of an enamel sample from a Leica DVM6 digital microscope, Mag. = 60× (**f**) and enamel apposition thickness measurements taken on one of the samples, Mag. = 120× (**g**).

DNA was extracted using the Sherlock AX kit (A&A Biotechnology, Gdańsk, Poland) according to the manufacturer’s protocol (see Figure 6a). The DNA sample underwent 32 RT-PCR cycles using TaqMan™ Genotyping Master Mix (ThermoFischer Scientific, Waltham, MA, USA) at concentrations of 2.5 μL per reaction and 0.125 μL per reaction, along with TaqMan SNP Genotyping Assays (ThermoFischer Scientific, Waltham, MA, USA). The total volume was 5 μL per reaction, with 1 μL of DNA in each. Genotyping of rs4694075 (*AMBN*) and rs2337359 (*TUFT1*) was performed using the LightCycler 480 System (Roche Diagnostics, Basel, Switzerland) (see Figure 6b,c).

Subsequently, the patients were grouped according to their genotype. Three groups of patients with the following genotypes were created for the *AMBN* polymorphism (rs4694075): C/C, C/T*, and T/T** (the asterisk signifies the presence of the T allele, which, according to the literature, is associated with caries predisposition). The patients were divided into three groups based on their genotypes for the *TUFT1* polymorphism (rs2337359): TT, C/T*, and C/C** (here, an asterisk indicates the presence of the C allele, which is associated with caries predisposition). The results from the studied parameters were then compared across these groups.

#### 4.5.2. Preparation of Teeth for Material, Chemical Composition, and Structure Analysis

The procedure for preparing the tooth samples was carried out in accordance with ISO 11609:2010 (International Organisation for Standardisation, Dentistry–Dentifrices–Requirements–Test Method and Marking) [19]. The crowns of the teeth, stripped of their roots, were cut mesio-distally using an IzoMet 1000 precision slow-speed diamond saw (Buehler Ltd., Lake Bluff, IL, USA). Each specimen was then embedded in epoxy resin with a partially exposed enamel surface (see Figure 6e). The surface was sanded with waterproof silicon carbide paper (Struers, Erkrath, Germany) at 320, 400, and 600 grit and polished at 1000 and 2000 grit using a Teragmin semi-automatic grinder–polisher (Struers GmbH, Willich, Germany).

#### 4.5.3. Teeth Chemical Composition Analysis

The chemical composition of the tooth surface was analysed using energy-dispersive X-ray spectroscopy (EDS) with a field emission scanning electron microscope (FE SEM ULTRA Plus, Carl Zeiss NTS GmbH, Oberkochen, Germany). A specific area of the sample surface is exposed to a concentrated electron beam, enabling the elemental composition to be determined [20,21]. For image analysis, the Smart SEM^®^ V05.05 operating software was used. The focus was placed on the calcium content of each enamel sample. Only teeth free of defects were selected for measuring these parameters (17 patients qualified for chemical composition analysis).

#### 4.5.4. Microhardness Analysis of the Enamel

The microhardness analysis was carried out using a Duramin-48 microhardness tester (Struers GmbH, Germany) and the Vickers method (load 25 g, time 5 s). Five indentations were made on each enamel sample, 100 µm apart along the enamel prisms. The area of indentation was used to calculate the hardness values [22,23] (see Figure 6d).

#### 4.5.5. Thickness of Enamel Apposition Layers

Enamel apposition was determined by measuring the distance between two adjacent Retzius striae at 120× magnification using a Leica DVM6 digital microscope (Leica Microsystems, Wetzlar, Germany). The microscope software, LAS X.next Version 5.2.2., enabled precise measurements with an accuracy of 0.01 μm. Five measurements were taken from the buccal or lingual side of each tooth sample at half the enamel thickness (see Figure 6f,g).

### 4.6. Statistical Analysis

The normality of the data distribution for the investigated parameters was assessed using the Shapiro–Wilk test, which revealed that the data were not normally distributed. Consequently, non-parametric statistical methods were employed. Measured teeth parameters were compared between genotype groups using the Kruskal–Wallis test for microhardness, enamel deposition thickness analyses, and the effect of SNP variants on the DMFT index. Following this, the Mann–Whitney U test with Bonferroni correction was used to determine which groups differed significantly. The correlation between enamel calcium content and the DMFT score was examined using Spearman’s rank correlation test. A *p*-value < 0.05 was considered statistically significant, and all analyses were conducted using the statistical program Statistica Version 14.3.0. Figures for publication were created with Biorender.com software.

## 5. Conclusions

1. The results of our study suggest that genetic variability in genes responsible for enamel formation may have a greater impact on the dynamics of demineralisation and remineralisation and interactions between enamel surface quality and the oral environment than on internal structural disorders or reduced enamel hardness.

2. The C/C genotype of the *TUFT1* rs2337359 polymorphism appears to be associated with increased susceptibility to dental caries, as measured by the DMFT index, suggesting its role in the aetiology of dental caries in the study population.

3. Reduced enamel calcium content is strongly associated with an increased risk of caries (higher DMFT index). This finding highlights the importance of monitoring calcium levels in enamel as an indicator of caries risk and suggests that targeted dental care is needed for this group of patients. The care procedure should consist of intensive demineralisation control algorithms using LIF and RVG, as well as remineralisation cycles to increase hydroxyapatite saturation with calcium phosphates, and the use of bioadhesive fluoride delivery systems.

## Data Availability

The raw data supporting the conclusions of this article will be made available by the authors on request.

## Abbreviations

The following abbreviations are used in this manuscript:

AMBN	gene encodes ameloblastin, a protein governing enamel development
API	approximal plaque index
DNA	deoxyribonucleic acid
DMFT	decayed, missing, filled permanent teeth index
EDS	energy-dispersive spectroscopy
FE SEM	field emission scanning electron microscopy
LIF	laser-induced fluorescence
RVG	radiovisiography
SNPs	single-nucleotide polymorphisms
TUFT1	gene encodes tuftelin 1, a protein governing enamel development
